# Metabolite Transformation and Enzyme Activities of Hainan Vanilla Beans During Curing to Improve Flavor Formation

**DOI:** 10.3390/molecules24152781

**Published:** 2019-07-31

**Authors:** Yingying Cai, Fenglin Gu, Yinghua Hong, Yonggan Chen, Fei Xu, Kejing An

**Affiliations:** 1 Spice and Beverage Research Institute, CATAS, Wanning 571533, China; 2 College of Food Science and Technology, Huazhong Agricultural University, Wuhan 430070, China; 3 National Center of Important Tropical Crops Engineering and Technology Research, Wanning 571533, China; 4 Hainan Provincial Engineering Research Center of Tropical Spice and Beverage Crops, Wanning 571533, China; 5 College of Tropical Biology and Agronomy, Hainan Tropical Ocean University, Sanya 572022, China; 6 Sericulture and Agri-Food Research Institute Guangdong Academy of Agricultural Sciences/Key Laboratory of Functional Foods, Ministry of Agriculture/Guangdong Key Laboratory of Agricultural Products Processing, Guangzhou 510610, China

**Keywords:** metabolites, vanilla flavors, vanillin precursors, enzyme activity

## Abstract

This paper compares the differences in metabolites of vanilla beans at five different curing stages. Key vanilla flavors, vanillin precursors and main enzymes during the curing process of Hainan vanilla beans were also analyzed. Hundreds of metabolites were detected based on metabolic analyses of a widely targeted metabolome technique, compared with blanched vanilla beans (BVB), sweating vanilla beans (SVB) and drying vanilla beans (DVB), the total peak intensity of cured vanilla beans (CVB) is on the rise. The score plots of principal component analysis indicated that the metabolites were generally similar at the same curing stages, but for the different curing stages, they varied substantially. During processing, vanillin content increased while glucovanillin content decreased, and vanillic acid was present in sweating beans, but its content was reduced in drying beans. Both *p*-hydroxybenzaldehyde and *p*-hydroxybenzoic acid showed the maximum contents in cured beans. Ferulic acid was mainly produced in drying beans and reduced in cured beans. *p*-coumaric acid increased during the curing process. Vanillyl alcohol in drying beans (0.22%) may be formed by the hydrolysis of glucoside, whose conversion into vanillin may explain its decrease during the curing stage. β-Glucosidase enzymatic activity was not detected in blanched and sweating beans, but was observed after drying. Peroxidase activity decreased during curing by 94% in cured beans. Polyphenol oxidase activity was low in earlier stages, whereas cellulase activity in processed beans was higher than in green beans, except for cured beans. This study contributes to revealing the formation of flavor components and the biosynthesis pathway of vanillin.

## 1. Introduction

Vanilla flavor has been the focus of many research groups for many years. Natural vanilla flavor is a complex mixture of more than 200 flavor components and is obtained from cured beans of *Vanilla planifolia* and *V. tahitensis* [1]. These form the highest-priced flavor ingredient in food (60%), cosmetics (33%) and aromatherapy (7%) [2,3]. Mature green vanilla beans are flavorless, the characteristic vanilla flavor in beans is formed only by a careful curing process that results in vanillin, vanillic acid and other flavor compounds [4].

Among these odor-active compounds, vanillin is the greatest contribution to the aroma of beans [5]. Nevertheless, vanillin content is usually ca. 1–2.5% *w*/*w* in cured beans. The annual global sales of vanillin have reached more than 15,000,000 kg in 2010, less than 1% of this value was obtained from vanilla beans [6,7]. Therefore, the synthesis pathway of vanillin has attracted much research interest, and different pathways have been suggested [6,8,9,10,11]. However, the synthesis pathway of vanillin in the beans has not been conclusive as hitherto demonstrated [11,12,13,14,15]. Improvement of natural vanillin production remains a major obstacle [16].

Many compounds were involved in the formation of vanillin. Previous research showed that vanillin and vanillic acid were derived from ferulic acid via radiolabelling methods [17]. Funk and Brodelius suggested a more complex route in which vanillin was formed from caffeic acid [8,9,10]. With the development in analytical chemistry, increasing compounds from other plants have been identified as precursors of vanillin, such as *p*-coumaric acid [18], glucovanillin [19], l-phenylalanine [11] and vanillyl alcohol [20]. Gu et al. reported the results of comprehensive metabolomic fingerprinting of vanilla fruits prepared from the curing process and constructed seven pathways of vanillin biosynthesis, including four new pathways, namely, glucose, cresol, capsaicin and vanillyl alcohol pathways [21]. 

The influence of enzymes and microorganisms on flavor formation in the vanilla curing process has been revealed recently [19,22,23,24]. β-d-Glucosidase plays the most important role in the curing process. Chen et al. found that colonizing *Bacillus* isolates produced β-d-glucosidase, which mediated glucovanillin hydrolysis and influences flavor formation [25]. In addition, researchers have suggested that enzymes such as polyphenol oxidases, peroxidases and proteases contribute to flavor development during curing of *V. planifolia* beans [26].

The main analytical techniques used in metabolomics analysis are nuclear magnetic resonance (NMR) spectroscopy, gas chromatography–mass spectrometry (GC–MS) and liquid chromatography–mass spectrometry (LC–MS). These techniques are suitable for the analysis of different concentrations and various physicochemical properties of metabolites [27]. Previous research showed that 22 primary metabolites and 55 secondary metabolites were identified in wines by NMR and GC [28]. Schmidtke et al. made it possible to detect several hundred compounds by GC–MS in wine [29]. However, NMR is the least sensitive of the three techniques. In addition, GC–MS is susceptible to sample derivatization [30]. Therefore, more information can be obtained by LC–MS, which has high sensitivity and is capable of detecting various metabolites [31]. Palama’s research on quantification of compounds based on LC–MS and NMR analysis revealed that vanillin could reach 24% of total vanillin content after eight months of development in vanilla beans [32]. Earlier work indicates that the metabolic changes of vanilla beans and vanilla beans by LC–MS to elucidate the biosynthesis of vanillin. Seven pathways for the biosynthesis of vanillin were constructed [21]. 

In this paper, the differences in metabolites of vanilla beans at five different curing stages were assessed by combining a liquid chromatography–tandem mass spectrometry (LC–MS/MS) detection platform, a self-built database and multivariate statistical analysis. High-performance liquid chromatography (HPLC) was used to quantify the main vanilla flavors and vanillin precursors in vanilla beans during curing, and the activities of four enzymes that play an important role in curing were determined. We analyzed and interpreted fluctuations of these compounds to reveal the biosynthesis pathway of vanillin.

## 2. Results 

### 2.1. Change of the Metabolites 

**Figure 1:**
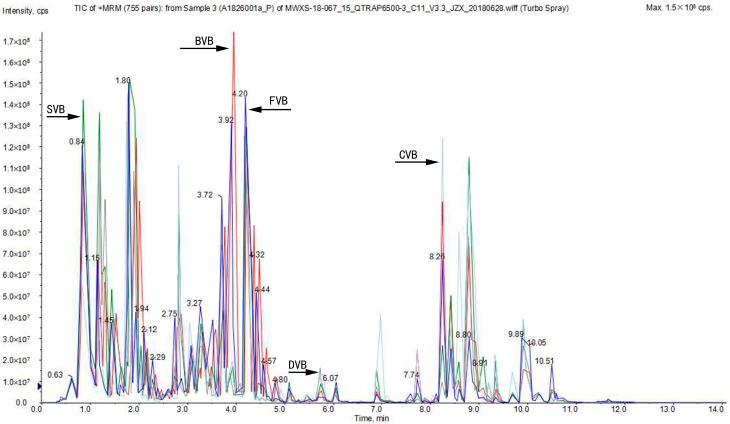
Liquid chromatography–tandem spectrometry (LC–MS/MS) base peak intensity (BPI) profiles of metabolites different processed vanilla beans (FVB (fresh vanilla bean): Blue, BVB (blanched vanilla bean): Red, SVB (sweating vanilla bean): Green, DVB (drying vanilla bean): Gray, and CVB (cured vanilla bean): Light blue).

The profiles were remarkably different at five different curing stages in vanilla. BVB (blanched vanilla bean) exhibited the most abundant metabolites, and the total peak area was the highest. Blanching destroyed the cell and tissue structure and a variety of precursors can be dissolved. The peak areas of SVB (sweating vanilla bean) and DVB (drying vanilla bean) showed a downward trend. Specifically, DVB was the lowest among the five different curing stages in vanilla. We speculated that they sweated at 55 °C for 6 h about six days and dried under the sun about one month, many metabolites were thus lost [33]. However, the total peak area of CVB (cured vanilla bean) was on the rise. This might be because the semi-finished beans treated in the early stage were sealed in a bag or placed in a closed box and stored for more than half a year. During this period, various chemical reactions and biochemical reactions, such as esterification, etherification and oxidation, occurred in the beans to form a unique aroma of vanilla. Therefore, large quantities of metabolites were produced [34,35]. Figure 1 shows clearly the changes in the relative content of metabolites at five different curing stages in vanilla.

### 2.2. Principal Component Analysis (PCA) 

**Figure 2:**
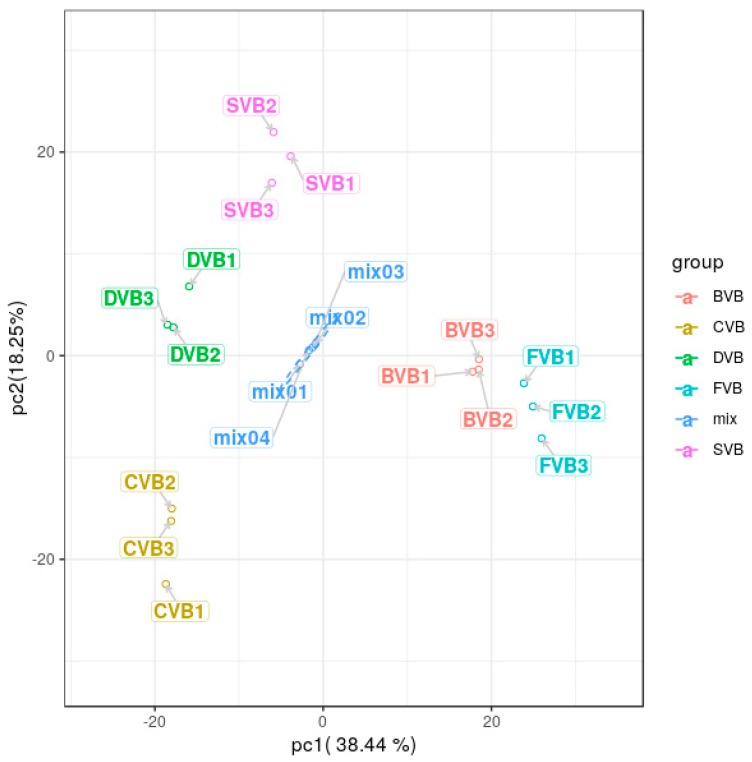
Principal component analysis (PCA) scores of vanilla beans during the curing process and the quality control sample. FVB: Fresh vanilla bean; BVB: Blanched vanilla bean; SVB: Sweating vanilla bean; DVB: Drying vanilla bean; CVB: Cured vanilla bean; mix: Mixture of extract of samples.

Principal component analysis (PCA) of samples (including quality control samples) provides a preliminary understanding of the overall metabolite difference between groups and the degree of variability between samples within the group. Wang et al. initially showed that the secondary metabolites in black sesame and white sesame were significantly different by PCA scores [36]. Yoshio et al. analyzed by PCA that the effects of cultivation conditions were greater than the species and sampling sites on metabolites in leaf lettuce [37]. Based on the Scree plot of cumulative eigenvalues (Figure 2), the first two principal components clearly separated all samples, and explained 56.69% of the total variation. In the PCA plot, the quality controlsamples grouped together, which indicated that the quality control samples had similar metabolic profiles and that the entire analysis was stable and repeatable. Vanilla beans from the same curing stages were clustered together, indicating that the metabolites were generally similar. On the contrary, vanilla beans from the different curing stages (FVB, BVB, SVB, DVB, CVB) were scattered, implying that their metabolites were quite different. Particularly, FVB showed significant differences from CVB. 

### 2.3. Change of the Main Vanilla Flavors

#### 2.3.1. Change of Vanillin

**Figure 3:**
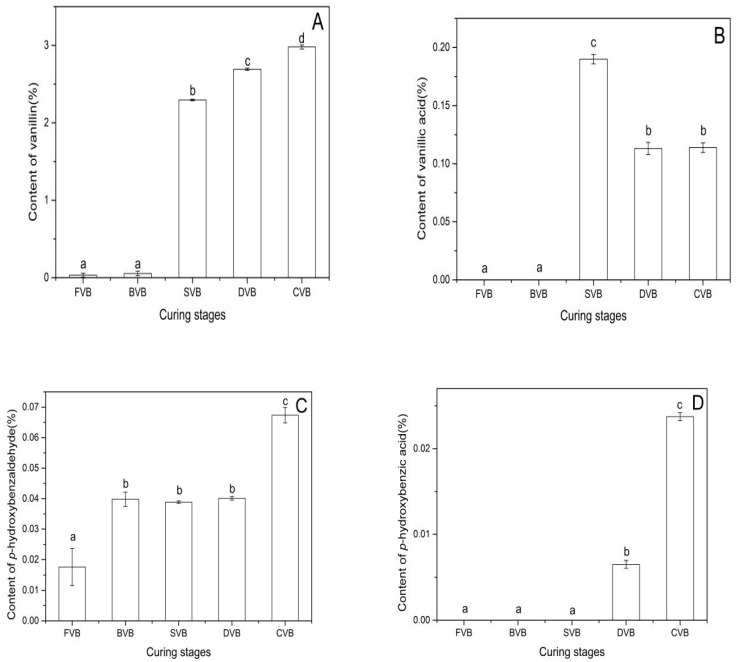
Amounts (% dw) of the main vanilla flavors in vanilla beans of the different curing stages: (**A**) Vanillin, (**B**) vanillic acid, (**C**) *p*-hydroxybenzaldehyde and (**D**) *p*-hydroxybenzoic acid. Bars represent the standard deviation of the three replicates. Data were subjected to Duncan’s test (*p* < 0.05). Different letters indicate quantities that are either statistically significantly different between different stages (a,b,c,d). FVB: Fresh vanilla bean; BVB: Blanched vanilla bean; SVB: Sweating vanilla bean; DVB: Drying vanilla bean; CVB: Cured vanilla bean.

The change of vanillin content at the curing stages was shown in Figure 3A. Vanillin was the most abundant odor compound in cured vanilla beans (1–3%) as previously reported in vanilla beans from different countries [5,35,38]. In the current study, vanillin content increased significantly after blanching, and the highest content (2.97%) was observed in cured beans.

The vanillin contents in green, blanched, sweating and drying beans were 0.03%, 0.05%, 2.30% and 2.69%, respectively. This result was in accordance with previous studies [25]. Most of the vanillin was produced in the period of sweating. Havkin-Frenkel et al. reported that oxidative and hydrolytic reactions might occur during this period [39]. Therefore, we established that vanillin was formed by the hydrolysis of glucovanillin or oxidation of vanillyl alcohol. According to Pardio et al. [35] and Havkin-Frenkel et al. [39], drying and curing may lead to loss in vanillin content. The yield of vanillin was more than what was detected in drying and curing beans.

#### 2.3.2. Change of Vanillic Acid

Dignum et al. found that vanillic acid concentration reached maximum (0.09%) in beans at nine days of curing and after eight days of sunning⁄sweating [34]. In this paper, vanillic acid was present in sweating vanilla beans (0.19%). This might be caused by the difference between blanching and sweating methods. It might have been formed by the oxidation of vanillin by peroxidases or the hydrolysis of glucovanillic acid [3,34,39]. The former might have occurred in the period of drying, whereas the latter might have occurred during sweating (Figure 3B). In the study of Kaur and Chakraborty, vanillic acid was either oxidatively decarboxylated to methoxyhydroquinone or reduced to vanillin and vanillyl alcohol. The yield of vanillin formed by vanillic acid decarboxylation was low [20]. Such phenomena may explain the decrease of vanillic acid content in the periods of curing and drying (Figure 3B).

#### 2.3.3. Change of *p*-hydroxybenzaldehyde 

*p*-hydroxybenzaldehyde is also considered as an indicator of quality and authenticity. The content in cured beans was affected by the method of curing, vanilla origin, etc., which ranged from 0.06% to 0.20% [40]. The *p*-hydroxybenzaldehyde contents were 0.017%, 0.040%, 0.039%, 0.040% and 0.067% in fresh, blanched, sweating, drying and cured beans, respectively (Figure 3C). *p*-hydroxybenzaldehyde in green beans was present mainly in its glucosidic form. The free form produced in the course of bean ontogeny may serve as the chemical defense of the maturing bean against herbivores and pests [11]. Dignum et al. reported that the affinity of β-glucosidase and glucosides of *p*-hydroxybenzaldehyde were in the same range as glucosides of vanillin. We inferred that the glucoside of *p*-hydroxybenzaldehyde was hydrolyzed in the period of blanching [19].

Gallage et al. reported the results of incubation with [14C]-*p*-hydroxybenzaldehyde in vanilla bean discs and demonstrated that *p*-hydroxybenzaldehyde was not an intermediate in vanillin biosynthesis [11]. This finding was backed in our study, in which *p*-hydroxybenzaldehyde content increased with the accumulation of vanillin. 

#### 2.3.4. Change of *p*-hydroxybenzoic Acid

The content of *p*-hydroxybenzoic acid in cured vanilla beans was 100 times lower than that of vanillin [40]. As shown in Figure 3D, the *p*-hydroxybenzoic acid contents in drying and cured beans were 0.006% and 0.026%, respectively. With the help of modern analytical techniques, glucoside of *p*-hydroxybenzoic acid and vanillic acid were identified in the green beans [41]. *p*-hydroxybenzoic acid mainly formed from the hydrolysis of its glycosides, which started in the period of drying.

### 2.4. Change of Vanillin Precursors

#### 2.4.1. Change of Glucovanillin

**Figure 4:**
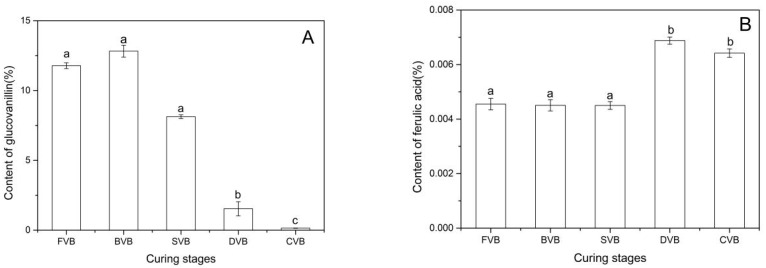

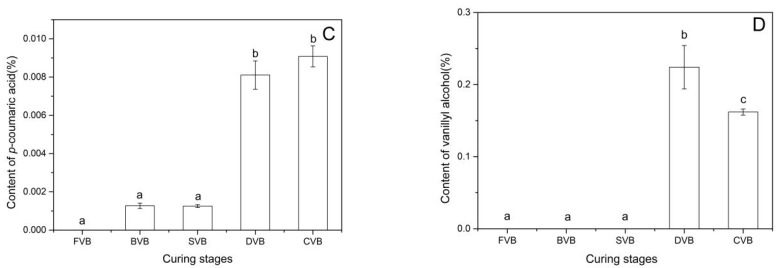
Amounts (% dw) of vanillin precursors in vanilla beans of the different curing stages: (**A**) Glucovanillin, (**B**) ferulic acids, (**C**) *p*-coumaric acid and (**D**) vanillyl alcohol. Bars represent the standard deviation of the three replicates. Data were subjected to Duncan’s test (*p* < 0.05). Different letters indicate quantities that are either statistically significantly different between different stages (a,b,c,d). FVB: Fresh vanilla bean; BVB: Blanched vanilla bean; SVB: Sweating vanilla bean; DVB: Drying vanilla bean; CVB: Cured vanilla bean.

As shown in Figure 4A, the glucovanillin contents decreased gradually during the curing process, which were 10.67%, 6.28%, 0.74%, 0.20% and 0.17% in fresh, blanched, sweating, drying and cured beans, respectively. After curing, a low amount of glucovanillin was left in the final vanilla beans. Thus, the hydrolysis of glucovanillin took place during the entire curing process. Dignum et al. reported that vanillin originated from β-glucosidase hydrolysis of glucovanillin [19]. In this paper, we found that the highest hydrolysis rate occurred during the period of sweating and drying. However, compared with the decrease of glucovanillin, the yield of vanillin was rather small (Figure 3A), especially in the drying period. This is probably because glucovanillin or vanillin was oxidized or reduced to other compounds, such as vanillyl alcohol [42]. In addition, the activities and quantity of enzymes also affected the vanillin liberation from glucovanillin. Pardio et al. reported the results of using hydrolytic enzymes to assist vanillin extraction and obtained higher vanillin content (4.38%) [35]. It is possible that the relative loss in bean weight resulted in the relative increase of glucovanillin during the period of blanching.

#### 2.4.2. Change of Ferulic Acid

The ferulic acid contents in fresh, blanched, sweating, drying and cured beans were 0.0046%, 0.0045%, 0.0045%, 0.0069% and 0.0064%, respectively. The results are shown in Figure 4B. Gallage et al. found that vanillin synthase could catalyze the direct conversion of ferulic acid and its glucoside into vanillin and its glucoside, respectively [11]. In addition, some microorganism played an important role in direct or indirect conversion of ferulic acid into vanillin [20,43]. As shown in Figure 4B, the conversion of ferulic acid into vanillin might have occurred during drying and curing. l-Phenylalanine and tyrosine produced ferulic acid via *p*-coumaric acid and caffeic acid [20,21]. It might be due to the increase in ferulic acid content during the drying period. Most of the ferulic acid in plants is bound to the plant cell wall, and enzymes that can cleave the bonds and release ferulic acid from microorganisms and plants have been found [44]. Therefore, treatment of beans with these enzymes during the drying or curing stages can promote the release of ferulic acid, thereby leading to the increase in vanillin production.

#### 2.4.3. Change of *p*-coumaric Acid

*p*-coumaric acid was not detected in fresh vanilla beans, but its content increased with the age of the bean after curing (Figure 4C). The highest *p*-coumaric acid content was observed in cured beans (0.009%), whereas 0.001% was in blanched and sweating beans. As previously mentioned, *p*-coumaric acid is an intermediate of the conversion of l-phenylalanine and tyrosine into ferulic acid. However, *p*-coumaric acid content did not change during sweating and increased in the drying period. Thus, we infer that the generation of *p*-coumaric acid and its conversion into caffeic acid were simultaneous in the period of sweating. A chain shortening of *p*-coumaric acid catalyzed by *p*-hydroxybenzaldehyde synthase produced *p*-hydroxybenzaldehyde [45]. Compared with the changes in *p*-coumaric acid and *p*-hydroxybenzaldehyde contents, the chain shorting occurred in the period of curing.

#### 2.4.4. Change of Vanillyl Alcohol

As shown in Figure 4D, vanillyl alcohol appeared in drying beans, and its content was 0.22%. After curing, it reduced to 0.16%. Vanillyl alcohol, which can be converted into vanillin by vanillyl alcohol oxidase directly, is also an intermediate of the conversion of cresol to vanillin [20]. Its conversion to vanillin might partly explain the decrease in its level during curing. Ranadive reported that vanillyl alcohol was present in glucosidic form in green beans, and the level of hydrolysis of glucosidic derivatives was affected by the curing and β-glucosidase activity [40]. The hydrolysis might occur during the drying period. 

### 2.5. Enzymatic Activities in Vanilla Beans 

#### 2.5.1. Change of β-Glucosidase 

**Figure 5:**
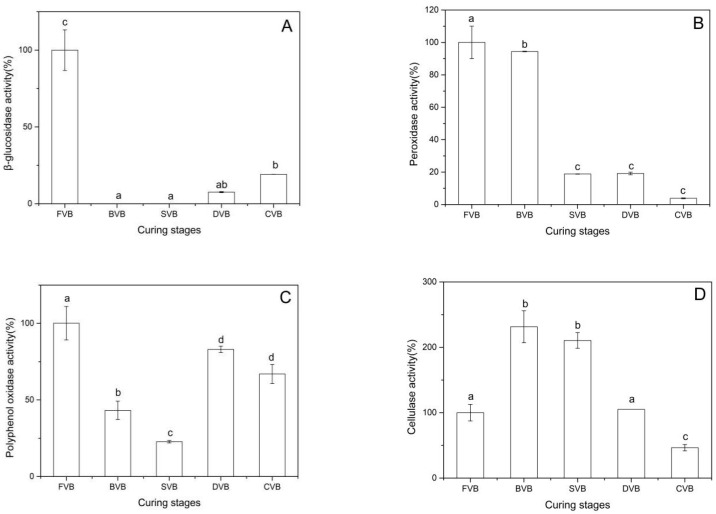
Enzymic activities in vanilla beans of the different curing stages: (**A**) β-glucosidase, (**B**) peroxidase, (**C**) polyphenol oxidase and (**D**) cellulase. Bars represent the standard deviation of the three replicates. Data were subjected to Duncan’s test (*p* < 0.05). Different letters indicate quantities that are either statistically significantly different between different stages (a,b,c,d). FVB: Fresh vanilla bean; BVB: Blanched vanilla bean; SVB: Sweating vanilla bean; DVB: Drying vanilla bean; CVB: Cured vanilla bean.

Figure 5A shows the results of the β-glucosidase analysis during curing. β-glucosidase showed the maximum activity in fresh beans and lost all activity during the period of blanching and sweating. According to Dignum et al., β-glucosidase showed a complete loss of activity after scalding at 70 °C and 80 °C [34]. Brillouet and Odoux studied β-glucosidase in mature green beans pretreated for 2 h at 50 °C, 55 °C and 60 °C and found that most of the β-glucosidase was heat-denatured by the pretreatment [46]. β-glucosidase appeared during drying, and the activity in cured beans was 25% of that in fresh beans. The microorganism that can produce the β-glucosidase appeared after sweating. Chen et al. reported that colonizing *Bacillus* isolates produced *β*-glucosidase, which mediated glucovanillin hydrolysis and influences flavor formation [25]. Various microorganisms colonizing the vanilla bean exhibited glycosidase activity and efficacy to convert ferulic acid to vanillic acid and related compounds [23,24]. Our study agrees well with these findings.

#### 2.5.2. Change of Peroxidase

Compared with *β*-glucosidase, peroxidase retained activity during the whole curing process (Figure 5B). The activity was 94% after blanching and 19% in the period of sweating and drying. In cured beans, peroxidase activity was reduced by 96% of that in fresh beans. Peroxidases play a role in the conversion of vanillin to vanillic acid during drying [26]. In addition, peroxidases also take part in Strecker degradation of amino acids by providing dicarbonyl compounds [26]. The peroxidase activity was absent from the trichomes and showed higher in mesocarp than in placentae [47]. Therefore, loss of moisture could result in the decrease of peroxidase activity.

#### 2.5.3. Change of Polyphenol Oxidase

As shown in Figure 5C, polyphenol oxidase activity was maximum in drying beans and remained at the same level in the subsequent curing process. At the blanching stage and sweating stage, it also maintained a certain level of activity, which was lower than that in drying and cured beans. Vanilla bean browning appears from the period of sweating but becomes heavier during the period of drying. Polyphenol oxidase in vanilla beans plays an important role in the oxidation of tyrosine, caffeic acids and other phenolic compounds, which may result in browning of vanilla beans [38,48]. This conclusion was confirmed in our study. In addition, polyphenol oxidase catalyzes the oxidation of vanillin, it may decrease vanillin content in the cured bean [48]. Therefore, we postulated that inhibition of polyphenol oxidase activity at curing stage helps improve the quality of vanilla beans.

#### 2.5.4. Change of Cellulase

Sreedhar et al. studied the impact of specific pretreatments on curing period of vanilla beans and found that cellulase activity in blanching beans significantly increased in comparison with the initial activity in green beans [22]. Cellulase activity of vanilla beans at different stages is shown in Figure 5D. In blanched beans, cellulase activity was at its maximum, which was 232% of that in fresh beans. Subsequently, activity decreased during the curing process and was only 47% in cured beans. The separation of flavor precursors and corresponding enzymes that catalyze their breakdown to final flavor components results in the absence of flavor. Cellulase plays an important role in cell wall degradation [38]. According to our study, this may occur in the period of blanching and sweating. We envisaged that up-regulation of the cellulase activity could enhance the flavor formation.

## 3. Materials and Methods

### 3.1. Materials

Glucovanillin was sourced from Sigma Chemical Company (St. Louis, MO, USA). Vanillin, vanillic acid, *p*-hydroxybenzaldehyde, *p*-hydroxybenzoic acid, ferulic acid, *p*-coumaric acid and vanillyl alcohol were obtained from Shanghai Aladdin Biochemical Technology Co., Ltd. (Shanghai, China). Methanol (MeOH), acetonitrile (ACN) and ethanol for LC–MS analysis (chromatographic purity grade) were purchased from Merck. Other chemicals for extraction analysis (analytical reagent grade) were purchased from Shanghai Chemical Reagent Co., Ltd. (Shanghai, China).

### 3.2. Curing Process and Sampling

Vanilla beans (*Vanilla planifolia* Andrews) were obtained from the Spice and Beverage Research Institute (Hainan, China) and cured by hot air processing [7]. Fresh beans were designated as FVB. After blanching (70 °C, 5 min), the beans were immediately obtained as BVB samples. Then, they underwent daily sun exposure for about 6 h to be heated. The beans were packed in cotton blankets in mahogany boxes for oven sweating at 55 °C for 6 h every day and cured for six days. After sweating, they were obtained as SVB samples. Finally, the beans were dried to reach a final moisture content of 30%. This procedure was repeated for 36 sunny days (not consecutively), and the beans were obtained as DVB samples. Finally, dry beans were conditioned in closed wooden boxes at room temperature for six months, and they were acquired as CVB samples. The samples were then frozen with liquid nitrogen and stored at −80 °C.

### 3.3. Sample Preparation and Extraction

The freeze-dried vanilla beans were crushed to powders by a mixer mill (MM 400, Retsch, Haan, German) for 1.5 min at 30 Hz, while 100mg powder was accurately weighted and extracted overnight at 4 °C with 1.0 mL 70% aqueous methanol. In order to increase the extraction rate, the samples were vortexed three times during this period. Following centrifugation at 10,000× *g* for 10 min, the supernatants were absorbed (CNWBOND Carbon-GCB SPE Cartridge, 250 mg, 3 mL; ANPEL, Shanghai, China) and filtrated (SCAA-104, 0.22 μm pore size; ANPEL, Shanghai, China) prior to LC–MS analysis. A quality control sample was prepared by equally blending all samples. During the assay, the quality control sample was run every 10 injections to monitor the repeatability of the samples under the same treatment method.

### 3.4. HPLC Conditions

The sample extracts were analyzed using an Liquid Chromatography-Electrospray Ionization-Tandem Mass Spectrometry (LC-ESI–MS/MS) system (HPLC, Shimpack UFLC Shimadzu CBM30A, Kyoto, Japan; MS, Applied Biosystems 6500 Q TRAP). The analytical conditions were as follows, HPLC: Column, Waters ACQUITY UPLC HSS T3 C18 (1.8 µm, 2.1 mm × 100 mm); Solvent system, water (0.04% acetic acid): Acetonitrile (0.04% acetic acid); gradient program, 95:5 *V*/*V* at 0 min, 5:95 *V*/*V* at 11.0 min, 5:95 *V*/*V* at 12.0 min, 95:5 *V*/*V* at 12.1 min, 95:5 *V*/*V* at 15.0 min; flow rate, 0.40 mL/min; temperature, 40 °C; injection volume: 2 μL. The effluent was alternatively connected to an ESI-triple quadrupole-linear ion trap (Q TRAP)-MS. After each injection, the needle was rinsed with 600 μL of weak wash solution (water/methanol: 90:10) and 200 μL of strong wash solution (methanol/water: 90:10). The samples were maintained at 6 °C during analysis.

### 3.5. ESI-Q TRAP-MS/MS

Linear ion trap (LIT) and triple quadrupole (QQQ) scans were acquired on a triple quadrupole-linear ion trap mass spectrometer (Q TRAP), API 6500 Q TRAP LC/MS/MS System, equipped with an ESI Turbo Ion-Spray interface, operating in a positive ion mode and controlled by Analyst 1.6 software (AB Sciex, Framingham, MA, USA). The ESI source operation parameters were as follows: Ion source, turbo spray; source temperature 500 °C; ion spray voltage (IS) 5500 V; ion source gas I (GSI), gas II (GSII), curtain gas (CUR) were set at 55, 60 and 25.0 psi, respectively; the collision gas (CAD) was high. Instrument tuning and mass calibration were performed with 10 and 100 μmol/L polypropylene glycol solutions in QQQ and LIT modes, respectively. QQQ scans were acquired as multiple reaction monitoring (MRM) experiments with collision gas (nitrogen) set to 5 psi. Declustering potential (DP) and collision energy (CE) for individual MRM transitions were obtained with further DP and CE optimization. A specific set of MRM transitions were monitored for each period according to the metabolites eluted within this period [49].

### 3.6. Metabolite Identification and Quantification

Qualitative analysis was performed on the data of the first-order spectra and the self-built database obtained by mass spectrum tests based on a self-built database MWDB (MetWare database) and the metabolite information common database. Among the metabolites, several substances have already been identified, and the repetitive signals of the adducts containing ions of K^+^, Na^+^, NH^4+^ and the mono-isotopic signal were removed during analysis. The repetitive signals of fragment ions with large molecular weights were also removed. For the structural analysis of metabolites, we referred to MassBank (http://www.massbank.jp/), KNAPSAcK (http://kanaya.naist.jp/KNApSAcK/), HMDB (http://www.hmdb.ca/), MoTo DB (http://www.ab.wur.nl/moto/), METLIN (http://metlin.scripps.edu/index.php) and other existing mass spectrum common databases. Metabolite intensity was conducted using MRM [50].

### 3.7. Determination of the Main Vanilla Flavors

The quantitations of vanillin, vanillic acid, *p*-hydroxybenzaldehyde and *p*-hydroxybenzoic acid were performed according to the method of Sinha et al. [51] with little modification. For extraction, the vanilla bean powders (2.00 g) from different curing stages were weighed in 50 mL Erlenmeyer flask. The mixture was shaken for 13 h at 50 °C after adding 50 mL of 70% ethanol–water solution. The samples were filtrated, and the solution volume was diluted to reach 50 mL. Samples were stored at 4 °C until they were needed for analysis. Each analysis on a sample was repeated twice. A mixture of 20% methanol and 80% acidified water was used for isocratic elution. This mixture was added at a flow rate of 1.0 mL/min. A UV detector was used at 280 nm, and the column temperature was maintained at 26 °C.

### 3.8. Determination of Vanillin Precursors

Glucovanillin content was determined using the method of Chen et al. [25]. Vanilla beans of different curing stages were finely crushed in liquid nitrogen (1 g) and placed in 100 mL Erlenmeyer flasks. Microwave irradiation (100 W, 20 min) was applied to assist the extraction after adding 100 mL of ethanol–water (70:30, *v*/*v*). The samples were passed through a 0.45 μm filter for HPLC analysis, which was equipped with a Zorbax Eclipse Plus C18 column (4.6 mm × 100 mm, 3.5 μm, Agilent, USA) to determine glucovanillin content. A mixture of 20% methanol and 80% acidified water was used for isocratic elution, and was added at a flow rate of 1.0 mL/min. The detection wavelength was set at 280 nm, and the column temperature was maintained at 26 °C. High performance liquid chromatography analysis conditions were performed as described above.

Ferulic acid, *p*-coumaric acid and vanillyl alcohol were extracted according to Sinha et al. with modifications [52]. For extraction, vanilla bean powder (2.00 g) was macerated in dehydrated ethanol for 12 h at room temperature. The extraction was repeated twice. The combined filtrate was concentrated under vacuum. Concentrated extract was redissolved in methanol (HPLC grade) to obtain 100 mL sample solution, which was passed through a 0.45 μm filter for HPLC analysis. Equipment of HPLC was the same as mentioned above. The UV detection wavelength was set at 280 nm, while the column temperature was maintained at 25 °C, and the injection volume of samples was 20 μL. The mobile phase was 100% of MeOH/ACN (1:1 *v*/*v*) and 0.2% acetic acid in water. For gradient elution, samples were separately eluted with 10%, 30%, 50%, 70%, 80% and 10% MeOH/ACN for 0.01, 8, 15, 20, 25 and 30 min, respectively. Equilibration was then conducted for 5 min. The flow rate of the mobile phase was 1 mL/min.

### 3.9. Determination of the Enzymatic Activities

Extraction and assay of β-glucosidase were performed according to the method described by Dignum et al. [26]. Two grams of vanilla beans at different curing stages were chopped into 1 cm pieces.BisTris-propane[1,3-bis[tris(hydroxymethyl)methylamino]-propane; BTP] buffer (150 mM) at 20 mL was used at pH 8.0. Ethylene Diamine Tetraacetic Acid (EDTA) and dithiothreitol (DTT) at 2 and 3 mM, respectively, were added to the buffer. Grinding was performed after adding 100 mg of PVPP. Extracts were centrifuged and then stored at −80 °C in the presence of 15% glycerol before enzyme analysis. The activity of β- glucosidase was determined at 30 °C according to Dignum et al. The reaction mixture contained 250 μL enzyme extract and 475 μL 2.1 mM *p*-nitrophenyl-β-d-glucopyranoside in 0.1 M phosphate buffer (pH 6.3). To inactivate the enzyme, 800 μL 1 M sodium carbonate was added after 30 min. The absorption was measured at 400 nm. The activity was calculated by using the formula ε400 = 18500/M cm, which was expressed by assuming that the mean of enzyme activity in fresh vanilla beans was 100%.

The extraction of peroxidase was the same as that of β-glucosidase. The activity was determined following the procedure explained by Dignum et al. [26]. A cuvette containing 0.3 mL guaiacol (40 mM) and 0.6 mL hydrogen peroxide (1.5 mM) in McIlvaine buffer (pH 3.8) were prepared. With addition of the 200 μL enzyme extracts, the mixture was reacted for exactly 3 min, where the absorbance was measured at 470 nm (ε470 = 26600/M cm). The activity was expressed by assuming that the mean enzyme activity in fresh vanilla beans was 100%.

Polyphenol oxidase activity was assayed using the method described by Malik and Singh [53]. The extraction involved chopping of beans into 1 cm pieces, of which 2 g was extracted with 20 mL of 0.1 M phosphate buffer (pH 6.5) and ground for homogenization. The extract was centrifuged at 75,000 g for 20 min. For the assay:1.5 mL catechol (0.01 M) prepared with 0.1 M phosphate buffer (pH 6.5) was added to 1 mL enzyme extract. After blending, the absorbance was measured at 495 nm, and the data was recorded in each case for 3 min at 30 s intervals. The activity was expressed by assuming that the mean enzyme activity in fresh vanilla beans was 100%.

Cellulase was performed according to the procedure of Sreedhar et al [4]. For extraction, 2 g of vanilla beans at different stages was chopped into 1 cm pieces and extracted at 4 °C with 20 mL sodium citrate buffer (pH 5). The extract was centrifuged twice at 5000 g for 15 min and the supernatant was used as the enzyme source. Cellulase activity was determined by incubating 0.5 mL of 0.1% (*w*/*v*) carboxymethylcellulose sodium (CMC) and 0.25 mL of sodium citrate buffer (pH 5) with 2.5 mL of enzyme extract for 1 h at 37 °C. The reaction was stopped by the addition of 1 mL NaOH (2M). After the addition of 1 mL DNS, the mixture reacted for 5 min in boiling water, and the absorbance was read at 490 nm. One unit of cellulase activity was the amount of enzyme that catalyzed the formation of 1 reducing group/min. The activity is expressed by assuming that the mean enzyme activity in fresh vanilla beans was 100%.

### 3.10. Statistical Analysis

Data were expressed as the mean ± standard deviation (SD). Analysis of variance (ANOVA) was performed, and comparisons of the means were carried out by least-significant difference (LSD) and Duncan. A value of *p* < 0.05 was considered statistically significant. Principal component analysis (PCA) was performed on the MetaboAnalyst 4.0 platform [54]. Data were analyzed by using Origin 9 (OriginLab Corporation, Northampton, MA, US) and SPSS Statistics 22 software packages (IBM Corporation, New York, NY, US).

## 4. Conclusions

In this study, the differences in metabolites of vanilla beans were compared at five different curing stages. Hundreds of metabolites were detected based on metabolic analysis of a widely targeted metabolome technique. Blanched vanilla beans (BVB) exhibited the most abundant metabolites, and its total peak area was the highest, whereas the peak areas of sweating vanilla beans (SVB) and drying vanilla beans (DVB) showed a downward trend. Compared with blanched vanilla beans (BVB), sweating vanilla beans (SVB) and drying vanilla beans (DVB), the total peak area of cured vanilla beans (CVB) was on the rise. The score plots of principal component analysis indicated that vanilla beans from the same curing stages were clustered together, revealing that the metabolites were generally similar. On the contrary, vanilla beans from the different curing stages were rather scattered, implying that the metabolites were quite different.

The contents of vanillin, vanillic acid, *p*-hydroxybenzoic acid and *p*-hydroxybenzaldehyde were also detected. The highest content (2.69%) of vanillin was observed in cured beans. Vanillic acid content was 10 times lower, whereas *p*-hydroxybenzoic acid and *p*-hydroxybenzaldehyde contents were 100 times lower than vanillin content in cured vanilla beans. *p*-hydroxybenzoic acid content increased, while vanillic acid decreased after the sweating stage. *p*-hydroxybenzaldehyde was produced mainly in the period of blanching and curing.

The precursors of vanillin were also determined by the high-performance liquid chromatography. The hydrolysis of glucovanillin occurred during sweating and drying period. Vanillic acid content was at its maximum in sweating beans, and its conversion into vanillin mainly occurred in the drying stage. The formation of vanillin and its glucoside from ferulic acid and its glucoside may take place in the periods of curing and blanching, respectively. By the detection of *p*-coumaric acid, the generation of *p*-coumaric may occur in the period of drying. Vanillyl alcohol may be formed by the hydrolysis of its glucoside during drying and converted into vanillin during curing.

*β*-Glucosidase lost all of its activity during blanching and sweating, but reappeared during drying. The activity in cured beans was 25% of that in fresh beans. Compared with β-glucosidase, peroxidase, polyphenol oxidase and cellulase maintained a certain level of activity in the developing vanilla bean. Peroxidase activity decreased during the curing process; activity was lowered by 94% of that in green beans. Polyphenol oxidase activity at the earlier curing stages was lower than that in drying and cured beans. Cellulase activity increased after blanching, with activity in blanching, sweating, and cured beans being 232%, 210% and 105% of that in fresh beans. It is beneficial to improve the quality of vanilla beans by controlling the activity of these enzymes at the appropriate curing stage.
